# Gestational diabetes and the incidence of diabetes in the 5 years following the index pregnancy in South Indian women

**DOI:** 10.1016/j.diabres.2007.06.002

**Published:** 2007-12

**Authors:** Ghattu V. Krishnaveni, Jacqueline C. Hill, Sargoor R. Veena, Suguna Geetha, Magudilu N. Jayakumar, Chitra L.S. Karat, Caroline H.D. Fall

**Affiliations:** a CSI Holdsworth Memorial Hospital, Mysore, Karnataka, India; b MRC Epidemiology Resource Centre, University of Southampton, Southampton, UK

**Keywords:** Gestational diabetes, Type 2 diabetes, Follow-up, India, Metabolic Syndrome

## Abstract

This study was carried out to examine the incidence of diabetes and the factors associated with this in a cohort of South Indian women 5 years after they were examined for gestational diabetes (GDM). Women (*N* = 630) whose GDM status was determined (Carpenter-Coustan criteria; GDM: *N* = 41) delivered live babies without major anomalies at the Holdsworth Memorial Hospital, Mysore. Of these, 526 women (GDM: *N* = 35) available for follow-up after 5 years underwent a 2-h oral glucose tolerance test and detailed anthropometry. Diabetes was determined using WHO criteria, and Metabolic Syndrome using IDF criteria recommended for south Asian women. The incidence of diabetes (37% versus 2%) and Metabolic Syndrome (60% versus 26%) was considerably higher in women with previous GDM compared to non-GDM women. GDM women who developed diabetes had lower gestational insulin area-under-the-curve (*P* = 0.05). They had larger waist-to-hip ratio, skinfolds, body mass index, and lower 30-min insulin increment at follow-up than other GDM women. In all, history of diabetes in first-degree relatives was independently associated with higher incidence of diabetes (*P* < 0.001). Our findings suggest high diabetes and cardiovascular risks in women with previous GDM. Follow-up of these women after delivery would provide opportunities to modify adverse lifestyle factors.

## 1 Introduction

Women with previous gestational diabetes (GDM) are at a higher risk of developing type 2 diabetes later in life, probably because both conditions share common risk factors [1,2]. Thus, early detection of the modifiable risk characteristics in GDM women may prevent or delay the disease process, thereby improving their quality of life.

In an earlier study, we measured glucose tolerance in a cohort of pregnant South Indian women [3]. The incidence of GDM (6.2%) was considerably greater than that reported earlier in Chennai (<1%) [4] and Kashmir in India (3.8%) [5], but less than that reported in another recent study from Chennai (16%) [6]. The women, who participated in the follow-up study examining the growth and cardiovascular risk factors in their children [7] 5 years after the index pregnancy were reviewed to study the incidence of type 2 diabetes in relation to their GDM status.

## 2 Materials and methods

### 2.1 Pregnancy

During 1997–1998, 830 women with no known history of diabetes, booking consecutively into the antenatal clinic of the Holdsworth Memorial Hospital (HMH) in Mysore, India, had a 100-g, 3-h, oral glucose tolerance test (OGTT) at 30 ± 2 weeks gestation; 785 women completed the OGTT [3]. Socio-economic status was assessed using the Kuppuswamy score, a questionnaire method, based on education, occupation and income [8]. Plasma glucose and insulin concentrations were measured as previously described [3]. GDM was diagnosed (*N* = 49, 6.2%) using the Carpenter and Coustan criteria [9]. Women's own consultant obstetricians managed their further clinical care. Of the 785 women, 630 who chose HMH for delivery gave birth to live babies without major anomalies and were included for further follow-up; 41 of these women had GDM and 12 of them were treated with insulin.

### 2.2 Follow-up

Further examination of these women was based on the follow-up of their offspring. Twenty-five children died between birth and 5 years, seven children were excluded after birth due to medical reasons, and 43 families either refused follow-up or moved away from Mysore. Accordingly 555 women were available for follow-up after 5 years. All willing, non-pregnant women, who had not been pregnant within the previous 6 months (*N* = 526) had a 2-h, 75-g OGTT; 524 women completed the test. Blood was taken fasting for plasma glucose, insulin, HDL-cholesterol and triglyceride concentrations, and 120 min after glucose load for plasma glucose and insulin. Women diagnosed with GDM at the index pregnancy (*N* = 35) also had a 30-min post-load sample. Weight; height; waist and hip circumferences; biceps, triceps, subscapular and suprailiac skinfold thicknesses were measured using standardized methods. Sum of skinfolds was obtained by adding individual skinfolds. Systolic (SBP) and diastolic blood pressures (DBP) were measured using an automated (CRITIKON, DINAMAP™ model 8100, FL, USA) BP monitor.

Glucose (glucose oxidase-peroxidase method), triglycerides (GPO-PAP method) and HDL-cholesterol (direct HDL-cholesterol method) were measured on an autoanalyzer (Abbott laboratories, USA), and insulin was measured using a time-resolved, fluoroimmunoassay (DELFIA) method (Southampton, UK) at the Diabetic Research Centre, KEM Hospital, Pune, India. Samples were stored at −80 °C until transfer to Pune. Only fasting samples were taken from one of two women known to have already developed diabetes; the other with previous GDM underwent a complete OGTT as she did not reveal the diagnosis until after the investigations.

Diabetes was defined as a fasting glucose concentration ≥7.0, and/or 120-min glucose ≥11.1 mmol/l (WHO criteria) [10]. Women were also classified as having diabetes if they had been diagnosed by a doctor as having diabetes since the index pregnancy. Impaired glucose tolerance (IGT) was a fasting glucose concentration <7.0 mmol/l and 120-min glucose ≥ 7.8 mmol/l, but <11.1 mmol/l. Impaired fasting glucose (IFG) was defined as a fasting glucose value of ≥6.1 mmol/l, but <7.0 mmol/l [10].

Metabolic syndrome was defined by the IDF criteria recommended for south Asian women [11]. Waist circumference ≥ 80 cm, and any two of the following: triglyceride ≥ 1.7 mmol/l; HDL-cholesterol < 1.29 mmol/l; SBP ≥ 130 or DBP ≥ 85 or having treatment for hypertension; fasting glucose ≥ 5.6 mmol/l or type 2 diabetes.

The hospital ethical committee approved the study, and informed verbal consent was obtained from the women.

### 2.3 Statistical methods

Insulin resistance was estimated using the Homeostasis Model Assessment equation (IR-HOMA) [12]. Insulin increment (a measure of insulin secretion) was derived using the formula: (30-min insulin-fasting insulin)/30-min glucose for all women during pregnancy, and for women with previous GDM at follow-up [13]. Using the OGTT data collected during pregnancy, area-under-the-curve values were calculated for glucose (GAUC) and insulin (IAUC) concentrations using the trapezoid rule [14]. GAUC, IAUC, IR-HOMA and insulin increment at the index pregnancy, and fasting glucose and triglycerides at follow-up were log-transformed to normality.

Differences in the prevalence of diabetes, IGT/IFG and Metabolic Syndrome between groups were analysed using chi-square tests. Differences in characteristics among women with diabetes, IGT/IFG and normal glucose tolerance (NGT) were examined using one-way ANOVA with linear trend tests. Logistic regressions were used to examine the predictors of diabetes at follow-up in all women.

## 3 Results

Two out of the 524 women (1 GDM at the index pregnancy, and 1 non-GDM woman who developed GDM in the subsequent pregnancy), who presented with symptoms since the index delivery had been diagnosed with type 2 diabetes by a doctor, and both were on treatment with oral sulfonylureas. At follow-up, we diagnosed diabetes in a further 12 women who had GDM in the index pregnancy (37%) and 7 non-GDM women (2%; OR = 35.5, 95% CI: 13.3–94.6, *P* < 0.001). The prevalence of IGT/IFG (31%; OR = 5.4, 95% CI: 2.3–12.9; *P* < 0.001) and Metabolic Syndrome (60%; OR = 4.4, 95% CI: 2.2–8.9, *P* < 0.001) was higher in women with previous GDM, compared with 15% and 26%, respectively in women who did not have GDM.

### 3.1 Women with GDM

Women with previous GDM who developed diabetes at follow-up had lower IAUC and higher GAUC during the index pregnancy compared to either IGT/IFG or NGT women (Table 1). They were also more insulin resistant and had lower 30-min insulin increment than non-GDM women who developed diabetes subsequently. Similar proportions of diabetic and IGT/IFG women were treated with insulin, while none of the NGT women had insulin during pregnancy. Women who had developed diabetes were more likely to be multiparous (parity ≥ 2) than NGT women, but the association was not statistically significant in these small numbers of women (Table 1).

At follow-up, GDM women who developed diabetes had larger body mass index (BMI), waist-to-hip ratio (WHR) and sum of skinfolds, were more insulin resistant, and had significantly lower insulin increment than IGT/IFG and NGT women (Table 1).

HDL-cholesterol concentrations were lower and the other indices of Metabolic Syndrome, and the prevalence of Metabolic Syndrome itself were higher in the women with diabetes at follow-up (Table 2). Diabetes was present in first-degree relatives in 92% (OR = 21.0, 95% CI: 2.3–192.8, *P* = 0.007) and Metabolic Syndrome was present in 85% of the diabetic women (OR = 6.6, 95% CI: 1.2–37.0, *P* = 0.03).

### 3.2 Women without GDM

In the index pregnancy, non-GDM women who later developed diabetes had higher glucose concentrations and a lower mean insulin increment than women who did not develop diabetes (Table 1). The proportion of multiparous women was higher in the group with diabetes.

At follow-up, women with diabetes were older, heavier (higher BMI), shorter, had larger WHR and sum of skinfolds, more insulin resistant and more likely to have a family history of diabetes than either IGT/IFG or NGT women (Table 1). Components of Metabolic Syndrome and the prevalence of Metabolic Syndrome (75%) were increased compared to IGT/IFG and NGT groups (Table 2).

Non-GDM women with diabetes appeared more obese and insulin resistant than respective GDM women, though these associations were not significant.

We have previously reported the effects of GDM in these mothers on the birth measurements of their newborns [3] and on the children's growth and glucose-insulin parameters at 5 years [7]. In the non-GDM group, newborns of women who developed diabetes had significantly greater mean ponderal index compared to those born to non-diabetic mothers (28.6 kg/m^3^ versus 24.9 kg/m^3^, *P* = 0.048). This difference remained significant after adjusting for maternal BMI and the child's sex (*P* < 0.001). There were no statistically significant differences in other birth measurements or in anthropometry, and glucose and insulin concentrations at 5-year follow-up in children born to non-GDM women with and without subsequent diabetes.

### 3.3 Predictors of diabetes at follow-up

Univariate logistic regression analyses in all women showed that the presence of GDM in the index pregnancy, higher current waist circumference, a positive family history of diabetes, older age, higher parity, higher GUAC, and fasting plasma glucose during pregnancy and lower insulin increment during pregnancy were risk factors for the development of diabetes (Table 3). Previous GDM, higher current waist circumference, and family history of diabetes remained significant risk factors after adjusting for other factors.

## 4 Discussion

We studied a cohort of South Indian women 5 years after they were investigated for the incidence of GDM as part of an earlier study [3]. The incidence of diabetes, IGT/IFG and Metabolic Syndrome was considerably higher in women who had GDM during the index pregnancy compared to non-GDM women. This was in spite of our inclusion criteria based on the offspring follow-up at 5 years after the index pregnancy resulting in a smaller group of GDM women available that may have resulted in an underestimation of the incidence rates.

We used the criteria of Carpenter and Coustan to diagnose GDM (because this was the test in routine clinical use in the hospital) and the WHO criteria to define diabetes at follow-up. Comparisons with NDDG and O'Sullivan's (Carpenter and Coustan) methods suggest that the WHO criteria are more sensitive in identifying GDM [15,16]. However, a study from Kashmir in India observed no significant difference in the prevalence of GDM between the WHO and the Carpenter and Coustan methods [5]. Thus, there is no reason to infer that the methods used in the study gave a markedly different estimation of GDM and diabetes incidence than if other methods had been used.

Women with previous GDM who later developed diabetes had evidence of lower insulin secretion in response to a glucose load during pregnancy compared to women who did not develop diabetes. They also had a strong family history of diabetes (>90%). Their high insulin resistance and BMI at follow-up were more in accordance with type 2 diabetes than type 1 diabetes. Women who develop GDM are said to be in a chronic insulin resistant state, and studies have observed higher insulin resistance in GDM women than in non-GDM pregnant women [17,18]. This coupled with deficient insulin secretion suggestive of deteriorating β-cell function underlies the development of GDM [17]. Our data shows that our GDM women were more insulin resistant, and also had lower insulin increment at the index pregnancy compared non-GDM women especially those who subsequently developed diabetes. Studies, including those using sophisticated techniques of measuring insulin secretion have shown that first phase insulin secretion relative to insulin resistance during pregnancy is an important predictor of later development of diabetes in GDM women [19,20]. Lower insulin at follow-up in our GDM women who developed diabetes indicate more impaired β-cell function, which may have led to the early progression to diabetes. Unfortunately we did not measure insulin increment in non-GDM women at follow-up; hence we do not know whether deteriorating β-cell function was a predictor of diabetes in them. However, these women were more obese and insulin resistant than diabetic women with previous GDM.

Higher parity was associated with an increase in the risk of diabetes at follow-up in our study. Pregnancy is a diabetogenic condition; gestational steroid hormones induce peripheral insulin resistance, thus increasing the stress on pancreatic beta cell function. Multiparity may therefore hasten deterioration to diabetes [21,22].

Our results are consistent with data from other countries that have confirmed an association between GDM and later type 2 diabetes [1,2,23,24]. In these studies, the incidence of diabetes varied from 2% to 70%, depending on the length of follow-up (6–8 weeks to 28 years) [1]. Many reported a high fasting glucose concentration during pregnancy as an important predictor of later diabetes [1]. High pre-pregnant weight/BMI and higher gain in weight/BMI post-partum were also risk factors [1,24]. Hyperglycaemia during the 4–16 weeks post-partum has also been shown to be a risk factor [23,24]. Thus, a post-partum OGTT may help to identify high-risk women who need more rigorous follow-up, and may provide scope to modify lifestyle factors. The American Diabetes Association recommends evaluation of glycaemic status for all GDM women 6 weeks after delivery [25]. However, very few of our study women had a follow-up examination after delivery (*N* = 7 out of 35). Generally low awareness regarding the risks of GDM and a sense of ‘complacency’ (among women themselves and their obstetricians) after the completion of pregnancy may be reasons for poor follow-up.

According to a recent estimate, about 10–31% of diabetes in parous women can be ascribed to previous GDM (population attributable ratio or PAR) [26]. As the PAR of an exposure increases with its prevalence in a population [26], the population impact of GDM may be large in countries like India. A recent report showed a high incidence of GDM [16%] among urban women in India [6]. Considering the large population, universality of marriage and childbearing in India, and high rates of GDM, a large proportion of diabetes among women may be detected early by proper screening for GDM and follow-up of GDM women post-partum.

Unfortunately, countries like India which are still struggling with poverty-related health risks, give little priority to detection and management of GDM and risk factors for type 2 diabetes. These issues are directly related to inadequacy of funds, specialist personnel and specialist laboratories, and lack of public awareness of the harmful effects of GDM. Our study highlights the need to educate the public in India about the long-term risks associated with GDM, at the same time stressing the need for better diagnostic and treatment protocols/facilities for gestational diabetes in public hospitals.

## Competing interest

None.

## Figures and Tables

**Table 1:** Characteristics of women with and without GDM during the index pregnancy

	GDM in index pregnancy				No GDM in index pregnancy				*P*^3^
	NGT (11)	IGT/IFG (11)	DM (13)	*P*^1^	*P*^2^	NGT (406)	IGT/IFG (75)	DM (8)	
Pregnancy									
GAUC (mmol)^a^	**1600.9** (1521, 1665)	**1610.5** (1550, 1782)	**1890.5** (1533, 2569)	0.07	<0.001	**1081.2** (982, 1196)	**1172.0** (1050, 1262)	**1106.7** (933, 1283)	0.001
IAUC/10^4^ (pmol)^a^	**7.9** (5.5, 10.7)	**8.7** (6.2, 11.2)	**4.9** (2.5, 7.6)	0.05	0.5	**5.1** (3.5, 7.3)	**5.0** (3.6, 7.2)	**4.3** (2.6, 6.4)	0.7
Fasting glucose (mmol/l)	**5.2** (5.0,5.8)	**5.3** (4.8, 5.7)	**6.1** (4.9, 6.9)	0.09	0.03	**4.5** (4.2, 4.7)	**4.6** (4.4, 4.9)	**4.3** (3.9, 5.0)	0.04
HOMA^a^	**1.9** (1.0, 2.8)	**2.1** (1.5, 3.3)	**2.1** (1.2, 3.6)	0.7	0.5	**1.1** (0.8, 1.6)	**1.3** (0.9, 1.8)	**1.0** (0.6, 2.1)	0.04
Insulin increment (pmol/mmol)	**21.6** (10.4)	**31.5** (15.4)	**18.2** (12.3)	0.5	<0.001	**54.4** (35.8)	**39.0** (24.0)	**34.7** (11.2)	0.008
Insulin therapy (*N*)^a^^,^^b^	**0** (0%)	**3** (27.3%)	**4** (30.8%)	0.07	–	–	–	–	–
Parity 2+ (*N*)^a^^,^^b^	**1** (9%)	**2** (18%)	**3** (23%)	0.2	0.04	**65** (16%)	**14** (19%)	**4** (50%)	0.2

Follow-up									
Age (years)^a^	**32.2** (28.0, 36.0)	**34.0** (30.0, 38.0)	**33.5** (29.5, 38.5)	0.5	0.03	**28.1** (25.0, 31.0)	**29.3** (27.0, 31.0)	**28.6** (27.3, 30.0)	0.01
BMI (kg/m^2^)	**23.6** (4.4)	**26.1** (3.0)	**26.7** (4.6)	0.08	<0.001	**23.2** (4.4)	**24.8** (4.4)	**28.9** (4.9)	0.3
Height (cm)	**153.9** (7.9)	**150.8** (6.5)	**152.6** (5.0)	0.7	0.02	**154.8** (5.3)	**153.3** (5.2)	**153.2** (5.1)	0.8
Waist–hip ratio	**0.87** (0.06)	**0.90** (0.08)	**0.93** (0.05)	0.04	<0.001	**0.88** (0.07)	**0.92** (0.07)	**0.95** (0.09)	0.5
Sum of skinfolds (mm)	**88.2** (39.9)	**122.8** (18.6)	**116.6** (41.5)	0.08	<0.001	**92.1** (39.9)	**108.2** (35.3)	**135.7** (38.0)	0.4
HOMA^a^	**1.8** (1.1, 2.6)	**2.4** (1.9, 3.5)	**3.6** (2.1, 5.7)	0.02	<0.001	**1.6** (1.1, 2.4)	**2.3** (1.6, 3.7)	**4.6** (3.6, 6.4)	0.4
Insulin increment (pmol/mmol)^a^	**30.5** (20.6, 34.8)	**27.7** (17.5, 38.9)	**6.9** (0.3, 11.6)	0.001	–	–	–	–	–
Family history (*N*)^a^^,^^b^	**5** (46%)	**3** (27%)	**12** (92%)	0.02	0.004	**101** (25%)	**27** (36%)	**5** (63%)	0.1

Values are mean (S.D.).

a Geometric mean (IQR) or *N* (%). *P* values derived using ANOVA for linear trend for *P*^1^ GDM group, *P*^2^ non-GDM group.

b *P* values derived using chi-square test for linear association. *P*^3^ for difference between GDM and non-GDM diabetic women.

**Table 2:** Indices of the Metabolic Syndrome at follow-up in women with and without GDM during the index pregnancy

	GDM in index pregnancy				No GDM in index pregnancy				*P*^3^
	NGT (11)	IGT/IFG (11)	DM (13)	*P*^1^	*P*^2^	NGT (406)	IGT/IFG (75)	DM (8)	

Waist circumference (cm)	**81.2** (10.0)	**85.8** (8.6)	**88.9** (9.2)	0.052	<0.001	**81.0** (11.5)	**86.2** (11.8)	**96.9** (16.0)	0.2
Fasting glucose (mmol/l)^a^	**5.3** (5.2, 5.8)	**6.0** (5.8, 6.1)	**10.6** (7.2, 14.3)	<0.001	<0.001	**5.2** (4.9, 5.6)	**6.1** (5.8, 6.4)	**7.5** (6.0, 9.5)	0.02
Triglycerides (mmol/l)^a^	**0.9** (0.8, 1.4)	**1.3** (0.7, 1.8)	**1.8** (1.2, 3.4)	0.003	0.004	**1.0** (0.7, 1.4)	**1.1** (0.8, 1.5)	**1.5** (0.9, 2.2)	0.8
HDL cholesterol (mmol/l)	**1.15** (0.1)	**1.14** (0.2)	**0.98** (0.2)	0.03	0.07	**1.14** (0.2)	**1.09** (0.2)	**1.11** (0.2)	0.7
Systolic BP (mmHg)	**106.7** (10.3)	**125.4** (19.1)	**121.1** (22.5)	0.08	<0.001	**107.2** (9.8)	**112.8** (12.1)	**117.7** (14.1)	0.3
Diastolic BP (mmHg)	**63.8** (8.3)	**73.5** (15.9)	**72.5** (11.5)	0.1	0.001	**64.8** (8.9)	**68.6** (10.0)	**68.2** (11.9)	0.6

†*N* (%) in each defining component of the Metabolic Syndrome									
Component 1	**6** (54.5%)	**8** (72.7%)	**11** (84.6%)	0.1	<0.001	**213** (52.5%)	**52** (69.3%)	**8** (100%)	0.4
2	**4** (36.4%)	**10** (90.9%)	**13** (100%)	<0.001	<0.001	**109** (26.8%)	**64** (85.3%)	**8** (100%)	–
3	**0** (0%)	**5** (45.5%)	**5** (38.5%)	0.05	0.01	**58** (14.4%)	**15** (20%)	**4** (50%)	0.5
4	**9** (81.8%)	**9** (81.8%)	**13** (100%)	0.2	0.7	**300** (74.6%)	**60** (80%)	**5** (62.5%)	0.04
5	**1** (9.1%)	**4** (36.4%)	**2** (15.4%)	0.8	<0.001	**8** (2%)	**10** (13.3%)	**1** (12.5%)	0.7

Metabolic syndrome (*N*)	**2** (18.2%)	**8** (72.7%)	**11** (84.6%)	0.001	<0.001	**75** (18.7%)	**44** (58.7%)	**6** (75%)	0.5

Values are mean (S.D.). *P* values derived using ANOVA or † chi-square test for linear association for *P*^1^ GDM group, *P*^2^ non-GDM group. *P*^3^ for difference between GDM and non-GDM diabetic women. Metabolic Syndrome components: (1) waist circumference ≥ 80 cm; (2) fasting glucose ≥ 5.6 mmol/l or type 2 diabetes; (3) plasma triglycerides ≥ 1.7 mmol/l; (4) plasma HDL cholesterol < 1.29 mmol/l; (5) systolic BP ≥ 130 or diastolic BP ≥ 85 mmHg or treatment for hypertension.

a Geometric mean (IQR) or *N* (%).

**Table 3:** Logistic regression analysis to test for the predictors of diabetes at follow-up in all study women

Predictors	Univariate			Multivariate^a^		
	Odds ratio	95% confidence interval	*P*	Odds ratio	95% confidence interval	*P*
GDM (yes/no)	**35.5**	13.3–94.6	<0.001	**53.0**	12.3–227.3	<0.001
Current height (cm)	**0.9**	0.9–1.0	0.1	**1.0**	0.9–1.1	0.7
Current waist circumference (cm)	**1.07**	1.03–1.1	<0.001	**1.1**	1.0–1.2	0.001
Family history (yes/no)	**11.5**	3.8–34.7	<0.001	**10.6**	2.9–39.2	<0.001
Socio-economic status (score)	**0.97**	0.9–1.1	0.5	**0.9**	0.8–1.0	0.2
Age (years)	**1.2**	1.1–1.3	0.001	**0.95**	0.8–1.1	0.6
Parity (*N*)	**1.6**	1.1–2.5	0.02	**1.8**	0.96–3.4	0.07
Gestational GAUC (mmol)	**1.005**	1.003–1.01	<0.001	–	–	–
Gestational fasting glucose (mmol/l)	**4.3**	2.2–8.3	<0.001	–	–	–
Gestational IAUC (pmol)	**0.999**	0.999–1.000	0.4	–	–	–
Insulin increment (pmol/mmol)	**0.97**	0.95–0.99	0.002	–	–	–

a All variables shown were included in the model simultaneously.
